# Knowledge, attitude, and practice towards face mask use among residents of Greater Chennai Corporation, India, March 2021

**DOI:** 10.3389/fpubh.2022.938642

**Published:** 2022-07-28

**Authors:** Ramya Nagarajan, Polani Rubeshkumar, Murugesan Jagadeesan, Mohankumar Raju, Manikandanesan Sakthivel, Sharan Murali, Muthappan Sendhilkumar, Kumaravel Ilangovan, Dineshkumar Harikrishnan, Vettrichelvan Venkatasamy, Parasuraman Ganeshkumar, Prabhdeep Kaur

**Affiliations:** ^1^Department of Noncommunicable Diseases, ICMR-National Institute of Epidemiology, Chennai, India; ^2^Department of Epidemiology, ICMR-National Institute of Epidemiology, Chennai, India; ^3^Greater Chennai Corporation, Chennai, India

**Keywords:** masks, COVID-19, compliance, public place, knowledge

## Abstract

**Background:**

Wearing a mask is one of the simplest ways to reduce the spread of COVID-19. Studies reported poor mask compliance in Greater Chennai Corporation, India. Hence, we described the knowledge, attitude, and practice regarding mask use among adults (≥18 years) in Greater Chennai Corporation, Tamil Nadu, India.

**Methods:**

We conducted a cross-sectional survey among residents of Greater Chennai Corporation in March 2021. We estimated the sample size to be 203 per strata (slum and non-slum). We used a simple random sampling technique to select 20 locations using a digital map in the slum and non-slum areas. After reaching the location chosen, we selected 10 consecutive households and one adult (≥18 years of age) from each household. We used a validated, semi-structured questionnaire for collecting data regarding knowledge, attitudes, and practices for mask use. We estimated proportions and 95% CI for key variables and compared the variables between slums and non-slums.

**Results:**

Of 430 participants included in the study, 51.4% were males. The mean (S.D.) age of the participants is 41.1 (14.6) years. The majority (86.7%) of the participants felt that wearing a mask helped in reducing the spread of coronavirus and the knowledge differed (*p*-value < 0.05) between the slum (81.4%) and non-slum (92.3%). Nearly half (46.5%) of the participants did not like being forced to wear the mask. About 63.9% of the participants reported the practice of mask use while going out which was similar across slums and non-slums.

**Conclusion:**

Although the knowledge regarding mask use was good among the public, the attitude was unfavorable. We suggest continuous reinforcement by spreading awareness and educating the community on the appropriate use of the mask.

## Introduction

Mask usage is considered one of the vital non-pharmacological interventions to control the spread of COVID-19 (1). It has been scientifically proven and recommended by global public health organizations to reduce the transmissibility and risk of infection due to SARS-CoV-2 (2–8). The World Health Organization (WHO), the U.S. Centers for Disease Control and Prevention (CDC), the Government of India, and numerous other government and public health agencies have recommended that people use masks in public settings when SARS-CoV-2, the virus that causes COVID-19, is being transmitted in the community (9–11). Early in the pandemic, before accumulating evidence that mask-wearing can reduce the spread of COVID-19, some countries with no history of the practice resisted adopting mask-wearing recommendations (12). In settings, mainly in Asia, where mask-wearing is common for people with even a minor cold, people were likelier to wear masks in public spaces, even without mandates.

As scientific understanding of COVID-19 has evolved, the importance of widespread use of masks has become clear, in part because of the transmission dynamics of the virus (13). People with COVID-19 are most infectious early in the disease, including before symptoms develop, and many people infected with COVID-19 never develop symptoms (14). The higher prevalence of asymptomatic infection makes wearing masks crucial, even among people who feel healthy (15). Promotion of mask-wearing should be part of a package of measures that includes handwashing, physical distancing, and interventions to reduce indoor exposures, find infected people and their contacts quickly, and provide rapid and supportive isolation and quarantine services (16).

Even with the increased necessity of face mask use, there is a wide variation in the knowledge, attitude and practice of mask use across the globe. While Tajvar et al. has documented poor knowledge with good attitude and practice toward mask use in Iran (17), Pramana et al. has documented satisfactory results in Indonesia (18). However, according to Azlan et al., Malaysia majority had a positive attitude toward mask use, but only half of the study participants were using face masks regularly (19). But a study by Tan et al. in China showed good compliance to mask use (20).

Although transmission risk is higher in indoor settings, the mask mandate was monitored, and authorized officials-imposed fines on non-compliant individuals, predominantly in public places such as traffic signals and streets (21). Our team previously conducted three surveys to monitor mask compliance in Chennai. We conducted the surveys in October 2020, December 2020, and March 2021. We selected outdoor public places for the first survey and added indoor settings in the second and third surveys. The compliance to appropriate mask use in three rounds was 28, 29, and 21% in the slums. The compliance was 36%, 35%, and 27% in non-slums after observing 3,600 individuals from 64 selected city streets (22). Additionally, indoor compliance was 11% in slums and 10% in the non-slums, while malls in the city showed the highest compliance for appropriate use of masks (57%) during the second round (22).

Although we documented poor compliance, there was limited understanding regarding attitudes and awareness in the population, which could influence their behaviors. Understanding the knowledge, attitude and practices (KAP) of the population will help the program managers and policy makers in strategizing the Information, Education and Communication (IEC) activities related to mask use. Based on our literature search there are no other studies on mask use from Greater Chennai Corporation or Tamil Nadu in community setting to determine KAP regarding mask use in India. Hence, we carried out this study to bridge this gap by estimating the knowledge and practices regarding the appropriate mask use and attitude toward wearing masks among adults in Greater Chennai Corporation, Tamil Nadu, India.

## Methods

### Study design and population

We conducted a cross-sectional survey among residents of Greater Chennai Corporation in March 2021. Chennai is a city in southern India governed by Greater Chennai Corporation. It is administratively divided into 15 zones covering 200 wards. This study was conducted in all the zones of Greater Chennai Corporation, covering both the slum and non-slum populations equally. The study population was adults ≥18 years of age residing within Greater Chennai Corporation.

### Sample size and sampling strategy

The sample size was estimated separately for the slum and non-slum populations. As per our previous survey (22), 70% of the population followed inappropriate mask use. With that we assumed that 70% of the study participants did not have the knowledge of appropriate mask use and estimated the sample size as 203 with 10% absolute precision, 95% confidence level, 20% non-response rate, and a design effect of 2. We included two strata, namely slum and non-slums. Hence sample size is 406 with 203 per strata.

All the zones under Greater Chennai Corporation limits were included in the study. We created a linelist of street separately for slums and non-slums. We randomly selected 20 sreets each from slums and non-slums. In each street we randomly selected a starting point using digital map. After selecting the starting point, we surveyed 10 consecutive households in the same street. We surveyed one adult (≥18 years of age) from each household available at home for the interview. If more than one eligible individual was available at home during the visit, we randomly selected one individual.

### Data collection

We reviewed the sample questionnaire on mask use from other studies and adapted it to the local setting (23). It was a validated, semi-structured questionnaire. We collected details on the sociodemographic profile, information on exposure to COVID-19, knowledge regarding masks used in different settings such as public places, public transport, attitude related to the mandatory mask use, and mask disposal practices. Most knowledge, attitude, and practice questions were asked on a Likert scale. However, the scales varied across the questions depending upon the nature of the question (Supplementary File 1). The data collection tool was translated into the vernacular language, pre-tested, and revised before the survey. We trained the field-level data collection team members and conducted simulation sessions to minimize the inter-observer variation. The data collection teams then interviewed the selected members face-to-face using the Open Data Kit (ODK) tool. COVID-19 appropriate behaviors were followed during the interview process.

### Operational definition

We defined “mask” as any cloth mask, medical mask, or N95 respirator worn over the face. “Public places” included both indoor and outdoor settings open to the public and did not have any entry restrictions (e.g., streets, bus stops, railway stations, grocery shops, vegetable shops, pharmacies, religious places, and apparel stores). Indoor Public places included places such as gyms, convention centers, and marriage halls. Outdoor places included places such as shops, bus stops, railway station, and religious places. “Workplace” included occupational settings open only to employees with limited access to the general public.

### Data analysis

We estimated the proportions with a 95% confidence interval (CI) using Stata version 16. We estimated the proportion of individuals who felt adopting appropriate mask use while in public places and at public transportation is needed. We also estimated the proportion of individuals who thought they shouldn't be forced to use masks and those who adopted appropriate mask disposal methods. We also used the chi-square test to compare the variables between the slum and non-slum populations. A *p*-value < 0.05 was considered statistically significant.

### Human subject protection

The approval for the study was obtained from the Institutional Ethics Committee, ICMR–NIE Chennai. Informed verbal consent was obtained from the study participants before collecting the data.

## Results

### Sociodemographic profile

Of 430 participants in our study, 221 were from slum areas and 209 from non-slum areas. Nearly half of the study participants (51.4%) were males (Table 1). The mean (S.D.) age of the participants was 41.1 (14.6) years (Slum: 42.4 (14.8) years; non-slum: 39.8 (14.3) years). Most of the study participants from the slums had secondary school education (34.3%), while those from the non-slums had graduate-level education (41.1%). Nearly 29.3% of the study participants were homemakers, followed by government employees (16.2%), and daily wage workers (8.8%), while rest of the population were self-employed, non-government employees, non-paid workers, students, retired personnel, and unemployed. Although the distribution of occupation did not vary between slums and non-slums (Table 1).

**Table 1:** Sociodemographic profile of the study participants, Greater Chennai Corporation, India, March 2021 (*N* = 430).

**Characteristics**	>**Slums (*N =* 221)**		>**Non-slums (*N =* 209)**		>**Total (*N =* 430)**		*p*-Value*(*X*^2^ test)
	**Frequency**	**Proportion (95% CI)**	**Frequency**	**Proportion (95% CI)**	**Frequency**	**Proportion (95% CI)**	
Gender
Male	114	51.5 (41.9–61.1)	107	51.2 (41.9–60.3)	221	51.4 (44.6–58.0)	0.953
Female	107	48.4 (38.8–58.0)	102	48.8 (39.6–58.0)	209	48.6 (41.9–55.3)	
**Education**							
Graduate and above	48	21.7 (13.9–32.2)	86	41.1 (31.1–51.9)	134	31.1 (24.1–39.1)	0.008^†^
Secondary school	76	34.3 (26.5–43.2)	61	29.1 (22.6–36.7)	137	31.8 (26.5–37.6)	
Primary school	58	26.2 (18.8–35.2)	45	21.5 (15.5–28.9)	103	23.9 (18.9–29.7)	
No education	39	17.6 (11.5–26.0)	17	08.1 (05.5–11.8)	56	13.0 (09.3–17.9)	
**Occupation**							
Government employee	26	11.7 (06.9–19.1)	44	21.0 (14.3–29.8)	70	16.2 (12.0–21.6)	0.236
Daily wages	22	09.9 (06.2–15.4)	16	07.6 (04.3–13.1)	38	08.8 (06.1–12.5)	
Home maker	62	28.0 (18.8–39.6)	64	30.6 (22.4–40.2)	126	29.3 (22.8–36.7)	
Others^‡^	111	50.2 (40.7–59.7)	85	40.6 (28.7–53.7)	196	45.5 (37.8–53.5)	

^*^P-Value < 0.05 was considered statistical significant.

*^†^Significant value.*

*^‡^Self-employed, non-government employees, non-paid workers, students, retired personnel, and unemployed. The bold values indicate the significant values*.

### Knowledge on mask use

A large proportion (86.7%) of respondents reported that mask-wearing reduces Coronavirus spread (Table 2). The knowledge was higher among respondents in non-slums compared to slums (92.3 vs. 81.4%, *p* < 0.05). The majority (87.6%) of the participants knew that masks should be worn while going to a public place, while 85.1% knew that masks should be worn while traveling in public transport. Nearly 80.9 and 83.9% of the participants knew that masks should be worn indoors and in public places. The indicators assessing the knowledge related to mask use in a public place and public transportation were similar among slums and non-slums (Table 2).

**Table 2:** Knowledge of mask use among the slum and non-slum population, Greater Chennai Corporation, India, March 2021 (*N* = 430).

**Characteristics**	>**Slums (*N =* 221)**		>**Non-slums (*N =* 209)**		>**Total (*N =* 430)**		
	**Frequency**	**Proportion (95% CI)**	**Frequency**	**Proportion (95% CI)**	**Frequency**	**Proportion (95% CI)**	p-Value^*^(X^2^ test)
**Does wearing a mask help to reduce the spread of the Coronavirus?**							
Yes	180	81.4 (73.2–87.5)	193	92.3 (87.1–95.5)	373	86.7 (81.5–90.6)	0.0054^†^
No	24	10.8 (07.0–16.3)	13	06.2 (03.2–11.5)	37	08.6 (05.9–12.3)	
Don't know/refused	17	07.6 (04.3–13.3)	3	01.4 (00.4–04.2)	20	04.6 (02.6–08.0)	
**Masks should be worn while going out of the home**							
Compulsory	190	85.9 (77.4–91.6)	187	89.4 (79.7–94.8)	377	87.6 (81.6–91.9)	0.7608
Optional	22	09.9 (04.8–19.3)	17	08.1 (03.4–18.0)	39	09.0 (05.2–15.2)	
Don't know	9	04.0 (02.0–07.9)	3	02.3 (00.4–11.4)	14	03.2 (01.5–06.6)	
**Masks should be worn while traveling in Public transport such as a bus etc.**,							
Compulsory	186	84.1 (75.6–90.1)	180	86.1 (75.7–92.5)	366	85.1 (78.8–89.7)	0.9326
Optional	26	11.7 (06.8–19.4)	21	10.5 (04.7–20.1)	47	10.9 (06.9–16.7)	
Don't know	9	04.0 (01.9–08.5)	8	03.8 (01.0–13.6)	17	03.9 (01.8–08.1)	
**Masks should be worn in indoor public spaces such as gyms, functions, marriage halls, etc.**,							
Compulsory	183	82.8 (72.6–89.7)	165	78.9 (68.2–86.7)	348	80.9 (73.8–86.4)	0.8147
Optional	28	12.6 (06.9–22.0)	33	15.7 (08.6–27.0)	61	14.1 (09.0–21.0)	
Don't know	10	04.5 (02.2–08.8)	11	05.2 (01.6–15.4)	21	04.8 (02.4–09.4)	
**Masks should be worn in all outdoor public spaces, such as shops, bus stops, etc.**,							
Compulsory	185	83.7 (74.4–90.0)	176	84.2 (72.7–91.4)	361	83.9 (77.0–89.0)	0.9775
Optional	25	11.3 (06.0–20.2)	24	11.4 (05.1–23.7)	49	11.4 (06.8–18.3)	
Don't know	11	04.9 (02.6–09.1)	9	04.3 (01.2–13.3)	20	04.6 (02.4–08.6)	

^*^P-Value <0.05 was considered statistical significant.

*^†^Significant value. The bold values indicate the significant values*.

### Attitude toward mask use

Nearly half (46.5%) of the participants felt they should not be forced to wear masks (Table 3). One-quarter (23.4%) of the participants reported that if they wear a mask in public, others will think they are affected by COVID-19. Nearly half of the subjects said masks disrupted breathing, caused overheating, and disturbed conversations. The proportion for attitude-related questions was similar among slums and non-slums. Out of 430 participants, 285 (66.2%) felt masks were not expensive.

**Table 3:** Attitude toward mask use among the slum and non-slum population, Greater Chennai Corporation, India, March 2021 (*N* = 430).

**Characteristics**	>**Slums (*N =* 221)**		>**Non-slums (*N =* 209)**		>**Total (*N =* 430)**		*p*-value*(*X*^2^ test)
	**Frequency**	**Proportion (95% CI)**	**Frequency**	**Proportion (95% CI)**	**Frequency**	**Proportion (95% CI)**	
**I shouldn't be forced to wear a mask**							
Agree	107	48.4 (37.9–59.0)	93	44.5 (33.7–55.8)	200	46.5 (38.9–54.2)	0.7591
Neither agree nor disagree	12	05.4 (03.4–08.4)	14	06.6 (03.4–12.4)	26	06.0 (04.0–08.9)	
Disagree	102	46.1 (34.8–57.8)	102	48.8 (37.0–60.6)	204	47.4 (39.2–55.8)	
**Everyone, including symptoms, should wear a cloth face covering if they leave their home to prevent possible transmission of the Coronavirus**							
Agree	141	63.8 (51.9–74.1)	162	77.5 (66.9–85.4)	303	70.4 (62.2–77.5)	0.1023
Neither agree nor disagree	32	14.4 (08.4–23.6)	26	12.4 (06.8–21.6)	58	13.4 (09.0–19.6)	
Disagree	48	21.7 (13.5–32.8)	21	10.0 (04.9–19.2)	69	16.0 (10.7–23.3)	
**I worry that if I wear a cloth face-covering out in public, other people will think I am infected with the Coronavirus**							
Agree	59	26.7 (18.3–37.1)	42	20.1 (12.6–30.5)	101	23.4 (17.5–30.7)	0.0944
Neither agree nor disagree	38	17.1 (11.5–24.8)	19	09.0 (05.3–14.9)	57	13.2 (09.5–18.1)	
Disagree	124	56.1 (44.7–66.8)	148	70.8 (57.8–81.0)	272	63.2 (54.4–71.2)	
**Face masks disrupt my breathing**							
Agree	127	57.4 (48.3–66.0)	108	51.6 (39.2–63.8)	235	54.6 (46.8–62.1)	0.1285
Neither agree nor disagree	2	00.9 (00.2–03.5)	12	05.7 (03.0–10.4)	14	03.2 (01.7–05.9)	
Disagree	92	41.6 (32.9–50.8)	89	42.5 (29.7–56.5)	181	42.0 (34.2–50.3)	
**Face masks cause me to overheat**							
Agree	106	47.9 (36.6–59.5)	99	47.3 (35.9–59.0)	205	47.6 (39.5–55.9)	0.8260
Neither agree nor disagree	14	06.3 (02.7–13.7)	18	08.6 (05.3–13.5)	32	07.4 (04.7–11.4)	
Disagree	101	45.7 (35.0–56.7)	92	44.0 (32.7–55.8)	193	44.8 (37.0–52.9)	
**Face mask disturbs my conversation with others**							
Agree	113	51.1 (41.7–60.4)	98	46.8 (35.9–58.1)	211	49.0 (41.8–56.3)	0.7416
Neither agree nor disagree	9	04.0 (01.8–08.5)	11	05.2 (02.6–10.0)	20	04.6 (02.8–07.6)	
Disagree	99	44.8 (34.3–55.7)	100	47.8 (36.0–59.8)	199	46.2 (38.3–54.4)	
**Face masks are unsafe because they force you to touch your face**							
Agree	76	34.3 (27.2–42.3)	46	22.0 (15.9–29.5)	122	28.3 (23.2–34.1)	0.1190
Neither agree nor disagree	35	15.8 (10.7–22.7)	40	19.1 (12.2–28.6)	75	17.4 (12.9–23.0)	
Disagree	110	49.7 (39.7–59.8)	123	58.8 (47.3–69.4)	233	54.1 (46.4–61.7)	
**Face masks are too expensive**							
Agree	66	29.8 (19.7–42.3)	52	24.8 (16.8–35.1)	118	27.4 (20.6–35.4)	0.6831
Neither agree nor disagree	12	05.4 (03.0–09.4)	15	07.1 (03.0–15.9)	27	06.2 (03.6–10.5)	
Disagree	143	64.7 (52.0–75.6)	142	67.9 (55.3–78.3)	285	66.2 (57.4–74.1)	

^*^P-Value <0.05 was considered statistical significant.

### Mask use practices

About 63.9% of the participants reported consistent mask use while going out (Table 4), while 58.8% used masks at their workplaces (Table 5). Most participants (86.7%) reported covering their chin, mouth, and nose while wearing the mask (Table 4). Handwashing after mask use was higher among non-slum respondents than among slum (47.4 vs. 26.2%, *p* < 0.05). Most participants disposed of their masks in a closed or public bin (91.2%).

**Table 4:** Practice of Mask use in Public Places among the slum and non-slum population, Greater Chennai Corporation, India, March 2021 (*N* = 430).

**Characteristics**	>**Slums (*N =* 221)**		>**Non-slums (*N =* 209)**		>**Total (*N =* 430)**		
	**Frequency**	**Proportion (95% CI)**	**Frequency**	**Proportion (95% CI)**	**Frequency**	**Proportion (95% CI)**	p-Value^*^(X^2^ test)
**How often do you wear a mask when you go out?**							
Always	132	59.7 (48.0–70.4)	143	68.4 (57.0–77.9)	275	63.9 (55.7–71.4)	0.4742
Most of the times	61	27.6 (18.4–39.1)	49	23.4 (16.0–32.9)	110	25.5 (19.3–32.9)	
Sometimes	15	06.7 (03.6–12.2)	11	05.2 (02.5–00.5)	26	06.0 (03.7–09.5)	
Rarely	13	05.8 (02.8–11.7)	6	02.8 (01.0–07.5)	19	04.4 (02.4–07.9)	
**What type of mask do you wear most of the time?**							
Cloth mask	157	71.0 (65.1–76.3)	136	65.1 (55.5–73.5)	293	68.1 (62.5–73.2)	0.3941
Medical mask	55	24.9 (19.7–30.8)	58	27.8 (21.1–35.5)	113	26.3 (21.9–31.0)	
N-95 masks/respirators	4	01.8 (00.5–05.6)	10	04.8 (02.5–08.8)	14	03.3 (01.8–05.7)	
Kerchief/ cloth fabric	5	02.2 (00.8–05.9)	5	02.3 (00.7–07.3)	10	02.3 (01.0–04.9)	
**How do you wear your mask most of the time?**							
Covering chin	3	01.4 (00.4–03.9)	6	02.9 (01.4–05.7)	9	02.1 (01.1–03.8)	0.0120^†^
Covering chin and mouth	31	14.0 (07.2–25.5)	7	03.3 (01.4–07.6)	38	08.8 (04.9–15.4)	
Covering chin, mouth and nose	180	81.4 (68.8–89.7)	193	92.3 (87.1–95.5)	373	86.7 (79.5–91.6)	
Below chin	7	03.1 (01.2–08.0)	3	01.4 (00.3–06.0)	10	02.3 (01.0–05.1)	
**Do you wash your hands before wearing the mask?**							
Daily/wash daily	72	32.6 (23.3–43.4)	85	41.0 (31.6–50.3)	157	36.5 (29.7–43.8)	0.1686
Once in 3 days	59	26.7 (17.8–37.8)	64	30.9 (22.1–40.6)	123	28.6 (22.2–35.9)	
Once in a week	52	23.9 (17.2–31.2)	42	20.2 (14.9–26.4)	94	22.1 (17.6–26.7)	
More than a week	38	17.1 (10.7–26.4)	18	08.6 (05.6–12.9)	56	13.0 (09.0–18.3)	
**How often do you touch the front side of your mask after wearing it?**							
Always	21	09.5 (05.3–16.3)	29	13.9 (09.6–19.5)	50	11.6 (08.4–15.8)	0.6064
Most of the times	63	28.5 (19.2–39.9)	64	30.6 (22.7–39.8)	127	29.5 (23.2–36.7)	
Sometimes	71	32.1 (24.8–40.4)	65	31.1 (23.8–39.4)	136	31.6 (26.3–37.4)	
Rarely	66	29.8 (21.2–40.1)	51	24.4 (17.7–32.5)	117	27.2 (21.5–33.7)	
**Do you wash your hands after removing the mask?**							
Always	58	26.2 (17.8–36.8)	99	47.4 (36.4–58.5)	157	36.5 (28.9–44.8)	0.0137^†^
Most of the times	55	24.9 (17.7–33.6)	43	20.6 (14.3–28.5)	98	22.8 (17.8–28.6)	
Sometimes	55	24.9 (19.1–31.6)	38	18.2 (12.2–26.1)	93	21.6 (17.2–26.7)	
Rarely	53	23.9 (15.4–35.3)	29	13.8 (09.0–20.7)	82	19.0 (13.7–25.9)	
**How frequently do you change/wash your mask?**							
Daily/wash daily	167	75.6 (65.4–83.5)	174	83.3 (75.1–89.0)	341	79.3 (72.7–84.6)	0.1597
Once in 3 days	24	10.9 (05.9–18.9)	23	11.0 (07.1–16.6)	47	10.9 (07.5–15.5)	
Once in a week	12	05.4 (02.9–99.4)	3	01.4 (00.3–06.0)	15	03.5 (01.9–06.3)	
More than a week	18	08.1 (03.9–15.9)	9	04.3 (02.3–07.8)	27	06.2 (03.7–10.4)	
**How do you dispose of the mask?**							
Into a public bin	63	28.5 (18.6–41.0)	71	34.0 (23.2–46.6)	134	31.2 (23.5–39.9)	0.1973
Collect in a bin	129	58.3 (46.7–69.1)	129	61.7 (49.8–72.3)	258	60.0 (52.1–67.4)	
Throw it in road	3	01.4 (00.4–03.9)	0	0.00 (00.0–00.0)	3	00.7 (00.2–02.1)	
Never dispose	26	11.7 (06.4–20.5)	9	04.3 (01.2–13.3)	35	08.1 (04.6–13.8)	

^*^P-Value <0.05 was considered statistical significant.

*^†^Statically significant*.

**Table 5:** Practice of Mask use at Workplaces among the slum and non-slum population, Greater Chennai Corporation, India, March 2021 (*N* = 238).

**Characteristics**	>**Slums (*N =* 132)**		>**Non-slums (*N =* 106)**		>**Total (*N =* 238)**		*p*-value*(*X*^2^ test)
	**Frequency**	**Proportion (95% CI)**	**Frequency**	**Proportion (95% CI)**	**Frequency**	**Proportion (95% CI)**	
**How often do you go to your workplace?**							
Daily	121	91.6 (82.4–96.2)	95	89.6 (80.6–94.7)	216	90.7 (84.8–94.5)	0.8315
Once in two-three days	7	05.3 (02.0–13.1)	7	06.6 (02.7–15.0)	14	05.8 (03.0–10.9)	
Once in a week	2	01.5 (00.3–05.9)	3	02.8 (00.9–08.4)	5	02.1 (00.8–05.0)	
More than a week	2	01.5 (00.3–06.1)	1	00.9 (00.1–06.5)	3	01.2 (00.3–03.9)	
**Do you wear a face mask in your workplace?**							
Always	70	53.0 (41.6–64.1)	70	66.0 (47.4–80.7)	140	58.8 (48.6–68.2)	0.1402
Most of the times	26	19.7(11.8–30.8)	23	21.7 (10.4–39.7)	49	20.5 (13.3–30.3)	
Sometimes	36	27.2 (19.5–36.6)	13	12.2 (06.9–20.7)	49	20.5 (14.9–27.6)	
**Do you share your food while eating at the workplace?**							
Always	18	13.6 (07.6–23.1)	2	01.8 (00.4–07.2)	20	08.4 (04.6–14.7)	0.0581
Most of the times	13	09.8 (03.8–22.7)	15	14.1 (06.8–27.1)	28	11.7 (06.5–20.3)	
Sometimes	101	76.5 (63.1–86.0)	89	83.9 (72.1–91.3)	190	79.8 (71.1–86.4)	
**Is your temperature checked daily at your workplace?**							
Always	28	21.2 (13.0–32.5)	38	35.8 (23.7–50.1)	66	27.7 (20.2–36.6)	0.2128
Most of the times	15	11.3 (05.2–23.0)	15	14.1 (05.1–33.5)	30	12.6 (06.6–22.5)	
Sometimes	89	67.4 (55.3–77.5)	53	50.0 (35.6–64.3)	142	59.6 (49.9–68.6)	
**Is hand sanitizer available at your workplace?**							
Always	51	38.6 (27.3–51.2)	61	57.5 (42.0–71.6)	112	47.0 (37.4–56.9)	0.1693
Most of the times	19	14.3 (08.0–24.3)	12	11.3 (05.1–23.2)	31	13.0 (08.1–20.0)	
Sometimes	62	46.9 (36.4–57.7)	33	31.1 (20.1–44.7)	95	39.9 (31.7–48.6)	
**Does your workplace encourage self-reporting of symptoms?**							
Yes	49	37.1 (25.7–50.1)	47	44.3(30.9–58.6)	96	40.3 (31.6–49.7)	0.5111
No	52	39.3 (27.0–53.2)	31	29.2 (18.4–43.1)	83	34.8 (26.1–44.6)	
Not sure	31	23.4 (13.4–37.7)	28	26.4 (12.6–47.0)	59	24.7 (15.8–36.6)	

^*^P-Value <0.05 was considered statistical significant.

Only one-third (34.6%) reported that physical distancing is strictly followed at their workplace (Table 6). A large proportion (59.4%) felt that maintaining physical distancing was difficult in the local context.

**Table 6:** Physical distancing practices among the slum and non-slum population, Greater Chennai Corporation, India, March 2021 (*N* = 430).

**Characteristics**	>**Slums (*N =* 221)**		>**Non-slums (*N =* 209)**		>**Total (*N =* 430)**		*p*-value*(*X*^2^ test)
	**Frequency**	**Proportion (95% CI)**	**Frequency**	**Proportion (95% CI)**	**Frequency**	**Proportion (95% CI)**	
**Is physical distancing being a follower strictly in your workplace?**							
Always	79	35.7 (26.9–45.6)	70	33.4 (24.7–43.5)	149	34.6 (28.2–41.6)	0.0743
Most of the times	24	10.8 (06.1–18.3)	33	15.7 (10.4–23.2)	57	13.2 (09.4–18.3)	
Sometimes	14	06.3 (03.7–10.4)	8	03.8 (01.6–08.6)	22	05.1 (03.2–07.9)	
Rarely	16	07.2 (03.2–15.2)	1	00.4 (13.5–27.5)	17	03.9 (01.7–08.6)	
Never	15	06.7 (04.0–11.1)	13	06.2 (03.3–11.1)	28	06.5 (04.4–09.5)	
Missing	73	33.0 (22.5–45.5)	84	40.1 (30.5–50.7)	157	36.5 (29.0–44.7)	
**Is physical distancing being implemented in the places you visit like markets, malls, and departmental stores?**							
Always	78	35.2 (24.1–48.3)	94	44.9 (33.5–56.9)	172	40.0 (31.6–48.9)	0.0820
Most of the times	52	23.5 (15.4–34.0)	51	24.4 (17.2–33.2)	103	23.9 (18.3–30.6)	
Sometimes	36	16.2 (09.3–26.9)	24	11.4 (06.6–19.0)	60	13.9 (09.3–20.2)	
Rarely	37	16.7 (09.7–27.2)	11	05.2 (02.6–10.1)	48	11.4 (06.9–17.4)	
Never	18	08.1 (04.5–14.2)	29	13.8 (06.6–26.5)	47	10.9 (06.1–17.5)	
Do you think maintaining physical distancing is difficult in our setting?							
Strongly agree	72	32.5 (24.7–41.5)	44	21.0 (15.8–27.4)	116	26.9 (02.5–08.4)	0.2562
Somewhat agree	71	32.1 (21.8–44.4)	69	33.0 (24.5–42.7)	140	32.5 (03.6–11.4)	
Neither agree nor disagree	10	04.5 (02.4–08.2)	15	07.1 (04.2–11.9)	25	05.8 (36.3–52.3)	
Somewhat disagree	20	09.0 (04.9–16.1)	15	07.1 (03.7–13.2)	35	08.1 (36.4–53.2)	
Strongly disagree	40	18.1 (10.8–28.7)	48	22.9 (14.9–33.6)	88	20.4 (14.7–27.7)	
Don't know/refused	8	03.6 (01.2–10.0)	18	08.6 (04.2–16.8)	26	06.0 (03.3–10.8)	

^*^P-Value <0.05 was considered statistical significant.

## Discussion

Most of the study participants knew that wearing a mask reduced the spread of COVID-19. The knowledge of mask use was higher in the non-slum population (92.3%) compared to the slum (81.4%). However, there was also a negative attitude toward wearing the mask (46.5%). Two-thirds (63.9%; slum: 59.7%; non-slum: 68.4%) of the participants reported consistent use of masks while going out, which was incompatible with our previous three surveys (slum: 28, 29, and 21%; non-slum: 36, 35, and 27%) based on observations in public places (22).

Our findings were consistent with studies from other low and middle-income countries, which reported high awareness about mask use (24, 25). A survey of 1,114 participants in Uganda reported knowledge of protection against COVID-19 by face masks among 86.4%. Another study conducted in Nepal among 381 individuals reported adequate knowledge of face mask use among 95.5% of the participants (24, 25). The knowledge was high in all the study settings, possibly due to frequent mentions of masks used in social media and mass media (26).

The attitude toward mask use was not encouraging, consistent with our previous surveys in the city that reported poor compliance (22). One in two study participants felt that they should not be forced to wear masks because masks interfered with breathing and speaking and caused overheating—a study conducted by Taylor et al. (27). Canada reported a negative attitude toward mask use. The respondents felt wearing a facemask was a hassle, looked ugly and silly, made other people uncomfortable and untrustworthy, and caused breathing difficulty and overheating (27).

We observed a disconnect between knowledge and attitude regarding mask use among the general public. Our study reported two-thirds of the study participants self-reported mask use while going to a public place, but this was not consistent with earlier surveys, which showed only 32% were using masks properly (22). Safe disposal was an important issue of concern with the increasing use of a mask during the pandemic (28). Disposal of the mask using a closed bin or a public bin was followed by more than two-thirds of the participants, according to WHO guidelines (29, 30). Whereas, previous study by Islam et al. in Bangladesh stated that only half of the study participants followed a safe disposal of the used mask (31). Mask use has also been an essential strategy in reducing the spread of infection in the workplace. Though WHO recommends using a mask by everyone at the workplace, only half of our study participants comply with it (32). We recommend strictly enforcing rules on mask use in public and workplaces.

Apart from mask use, physical distancing is an effective way of reducing the spread of infection in the community (1). WHO has recommended maintaining physical distancing in public and workplaces (33). The same is also adapted in India to prevent SARS-CoV-2 infection (34–36). Even from the previous influenza pandemic, several studies supported the social distanceing at workplace to prevent spread of infection (37). However, only one-third of the study participants followed physical distancing at the workplace. This could be possibly due to practical challenges in distancing at markets, workplaces, and slums in our setting. Therefore, masks will be more important in crowded cities, especially where many people come together in closed spaces.

This major strength of our study was that we surveyed a representative sample of respondents from the slum and non-slum population in a large metropolitan city in India. Hence, the results can be generalized to the slum and non-slum of a meteropolitan city in India. One of the limitations was we could not observe the study participants for mask use. Hence, the reported practice of mask use could be overestimated as it was based on self-reporting by the respondents. Hence, we recommend combining methods, including questionnaire-based surveys and observation-based studies, to understand the mask use. The second limitation was inter-observer variation during the data collection as multiple teams collected data simultaneously. However, we tried to minimize this error through training all the data collectors simultaneously and simulation of the interviews.

We conclude that the community knew the benefits of masks used in a large metropolitan city in India. However, the attitudes and practice were not satisfactory. We suggest continuous reinforcement by spreading awareness and educating on the appropriate use of the mask in the community using mass media. We also suggest addressing the misconceptions related to mask use such as difficulty in breathing, conversation, and overheating. We also recommend strict enforcement of regulations in public places and workplaces to contain the spread of COVID-19 in the community.

## Data availability statement

The raw data supporting the conclusions of this article will be made available by the authors, without undue reservation.

## Ethics statement

The studies involving human participants were reviewed and approved by Indian Council of Medical Research–National Institute of Epidemiology. Written informed consent for participation was not required for this study in accordance with the national legislation and the institutional requirements.

## Author contributions

RN: conceptualization, methodology, data collection, analysis, manuscript writing, and preparation of first draft. PK, PG, and MJ: conceptualization, methodology, and critical review and revision of the manuscript. VV and DH: data management and critical review and revision of the manuscript. KI and MSe: data collection, management of field activities,data analysis, and critical review and revision of the manuscript. SM and MSa: data collection, data analysis, and critical review and revision of the manuscript. MR: methodology, data collection, data analysis, and critical review and revision of the manuscript. PR: conceptualization, data collection, analysis, and preparation of first draft. All authors contributed to the article and approved the submitted version.

## Funding

This study was funded by the Intra-Mural Fund of ICMR–National Institute of Epidemiology, Chennai, India. The funders had no role in study design, data collection, analysis, publishing decisions, or manuscript preparation.

## Conflict of interest

Author MJ was employed by Greater Chennai Corporation. The remaining authors declare that the research was conducted in the absence of any commercial or financial relationships that could be construed as a potential conflict of interest.

## Publisher's note

All claims expressed in this article are solely those of the authors and do not necessarily represent those of their affiliated organizations, or those of the publisher, the editors and the reviewers. Any product that may be evaluated in this article, or claim that may be made by its manufacturer, is not guaranteed or endorsed by the publisher.
