# Supplementary material for: Knowledge, attitude, and practice towards face mask use among residents of Greater Chennai Corporation, India, March 2021
Source: Front Public Health. 2022 Jul 28;10:938642. doi: 10.3389/fpubh.2022.938642 (PMC9367688; doi:10.3389/fpubh.2022.938642)
Supplement: Supplementary file 1 [file Data_Sheet_1.PDF]

Supplementary file 1: Surveillance for mask compliance, Tamil Nadu, India

S. no	Question	Type of variable	Response	Remarks
Interview details				
1.	Date of interview	Date entry		
2.	Name of the interviewer	Text		
3.	Zone (For Chennai)	Nominal	a) Zone 1<br>b) Zone 2<br>c) Zone 3<br>d) Zone 4<br>e) Zone 5<br>f) Zone 6<br>g) Zone 7<br>h) Zone 8<br>i) Zone 9<br>j) Zone 10<br>k) Zone 11<br>l) Zone 12<br>m) Zone 13<br>n) Zone 14<br>o) Zone 15	
4.	Street/village code (pre-assigned)	Numeric		
5.	Locality/Area (e.g. Velachery)	Text		
Demographics				
6.	ID (auto assign)	Alpha numeric		
7.	Age (completed years)	Discrete		
8.	Gender	Nominal	1. Male<br>2. Female<br>3. Transgender	
9.	Education	Ordinal	1. Graduate & above<br>2. Secondary school<br>3. Primary school<br>4. No education	

10.	Occupation	Nominal	1. Govt employee<br>2. Non-govt employee<br>3. Daily wages<br>4. Self-employed<br>5. Non-paid<br>6. Student<br>7. Home maker<br>8. Retired<br>9. Unemployed (able to work)<br>10. Unemployed (unable to work)	
**COVID-19 status**				
11.	Have you been tested for COVID-19 so far?	Nominal	1. Yes<br>2. No	If the answer is "No", go to Q.no. 13
12.	Were you declared positive for COVID-19?	Nominal	1. Yes<br>2. No	
13.	Did any of the household members test positive for COVID-19?	Nominal	1. Yes<br>2. No	
14.	Did any of your relatives/friends test positive for COVID-19?	Nominal	1. Yes<br>2. No	
15.	Did anyone known to you (household members/friends/relatives) die of COVID-19?	Nominal	1. Yes<br>2. No	
**Awareness and Knowledge of COVID-19**				
16.	How would you rate your knowledge level on how to prevent spread of the Coronavirus, between 0 to 5, 0 being "No knowledge" and 5 being the Excellent knowledge	Ordinal	Likert 0 to 5	
17.	Please indicate what do you think as your level of risk for contracting coronavirus or COVID-19, between 0 to 5, 0 being "No risk" and 5 being the highest risk.	Ordinal	Likert 0 to 5	

18.	How are you practising the following steps to protect yourself from the Coronavirus?	Group of question		
19.	1. Physical distancing	Nominal	1. Always<br>2. Sometimes<br>3. Never	
20.	2. Staying confined within the home	Nominal	1. Always<br>2. Sometimes<br>3. Never	
21.	3. Non-essential travel	Nominal	1. Frequent<br>2. Sometimes<br>3. Never	
22.	4. Visiting family, friends in their homes	Nominal	1. Frequent<br>2. Sometimes<br>3. Never	
23.	5. Postponing/cancelling medical appointments, procedures or surgeries	Nominal	1. Always<br>2. Sometimes<br>3. Never	
24.	6. Inviting guests to home	Nominal	1. Frequent<br>2. Sometimes<br>3. Never	
25.	7. Allowing maid or people who come for household work into house	Nominal	1. Always<br>2. Sometimes<br>3. Never	
26.	8. Wearing a mask or face covering while moving outdoors?	Nominal	1. Always<br>2. Sometimes<br>3. Never	
27.	9. Wearing a mask or face covering while moving speaking with neighbours or guests?	Nominal	1. Always<br>2. Sometimes<br>3. Never	
28.	10. Carrying a hand sanitizer while going out of home to sanitize your hands	Nominal	1. Always<br>2. Sometimes<br>3. Never	

29.	11. Washing your hands immediately after entering home	Nominal	1. Always<br>2. Sometimes<br>3. Never<br>4. Not applicable (If never goes out home)	
Knowledge Masks				
30.	Does wearing a mask help to reduce the spread of the coronavirus?	Nominal	1. Yes, a lot<br>2. Yes, some<br>3. No, it does nothing<br>4. Don't know/refused	
31.	As far as you are aware, what is our current Government policy on wearing face coverings in each of the following places? Masks should be worn	Group of questions		
32.	1. While going out of the home	Nominal	1. Compulsory<br>2. Optional<br>3. Don't know	
33.	2. While travelling in Public transport such as a bus etc	Nominal	1. Compulsory<br>2. Optional<br>3. Don't know	
34.	3. In all indoor public spaces such as gyms, functions, marriage halls, etc.	Nominal	1. Compulsory<br>2. Optional<br>3. Don't know	
35.	4. In all outdoor public spaces, such as shops, bus stops, etc	Nominal	1. Compulsory<br>2. Optional<br>3. Don't know	
Proper mask usage				
36.	How often you wear mask when you go out?	Nominal	1. Always<br>2. Most of the times<br>3. Sometimes<br>4. Rarely<br>5. Never	**Skip to Q. No. 45 if the answer is "Never"**
37.	What type of mask you wear most of the times?	Nominal	1. Cloth mask	

			2. Medical masks<br>3. N-95 masks/respirators<br>4. Pollution mask<br>5. Kerchief/ cloth fabric	
38.	How do wear your mask most of the time?	Nominal	1. Covering chin<br>2. Covering chin and mouth<br>3. Covering chin, mouth and nose<br>4. Below chin	
39.	Do you wash hands before wearing the mask?	Nominal	1. Always<br>2. Most of the times<br>3. Sometimes<br>4. Rarely<br>5. Never	
40.	How often you touch the front side of your mask after wearing it?	Nominal	1. Always<br>2. Most of the times<br>3. Sometimes<br>4. Rarely<br>5. Never	
41.	Do you wash your hands after removing the mask?	Nominal	1. Always<br>2. Most of the times<br>3. Sometimes<br>4. Rarely<br>5. Never	
42.	How frequently you change/wash your mask?	Nominal	1. Daily/wash daily (applicable only for cloth mask)<br>2. Once in two-three days<br>3. Once in a week<br>4. More than a week<br>5. Never change	
43.	How do you dispose the mask?	Nominal	1. Into a public bin<br>2. Collect in a bin/cover and dispose into public bin	

			3. Throw it in road<br>4. Never dispose<br>5. Other ________________	
44.	How often you carry hand sanitizer with you?	Nominal	1. Always<br>2. Most of the times<br>3. Sometimes<br>4. Rarely<br>5. Never	
Attitude towards masks				
45.	**How strongly do you agree or disagree with the following?**			
46.	I shouldn't be forced to wear a mask	Nominal	1. Strongly agree<br>2. Somewhat agree<br>3. Neither agree nor disagree<br>4. Somewhat disagree<br>5. Strongly disagree<br>6. Don't know/refused	
47.	Everyone, including people who do not have symptoms, should wear a cloth face covering if they leave their home to prevent possible transmission of the Coronavirus.	Nominal	1. Strongly agree<br>2. Somewhat agree<br>3. Neither agree nor disagree<br>4. Somewhat disagree<br>5. Strongly disagree<br>6. Don't know/refused	
48.	I worry that if I wear a cloth face covering out in public, other people will think I am infected with the Coronavirus.	Nominal	1. Strongly agree<br>2. Somewhat agree<br>3. Neither agree nor disagree<br>4. Somewhat disagree<br>5. Strongly disagree<br>6. Don't know/refused	
49.	Face masks disrupt my breathing.	Nominal	1. Strongly agree<br>2. Somewhat agree<br>3. Neither agree nor disagree<br>4. Somewhat disagree	

			5. Strongly disagree<br>6. Don’t know/refused	
50.	Face masks cause me to overheat	Nominal	1. Strongly agree<br>2. Somewhat agree<br>3. Neither agree nor disagree<br>4. Somewhat disagree<br>5. Strongly disagree<br>6. Don’t know/refused	
51.	Face mask disturbs my conversation with others	Nominal	1. Strongly agree<br>2. Somewhat agree<br>3. Neither agree nor disagree<br>4. Somewhat disagree<br>5. Strongly disagree<br>6. Don’t know/refused	
52.	Face masks are unsafe because they force you to touch your face	Nominal	1. Strongly agree<br>2. Somewhat agree<br>3. Neither agree nor disagree<br>4. Somewhat disagree<br>5. Strongly disagree<br>6. Don’t know/refused	
53.	Face masks are too expensive.	Nominal	1. Strongly agree<br>2. Somewhat agree<br>3. Neither agree nor disagree<br>4. Somewhat disagree<br>5. Strongly disagree<br>6. Don’t know/refused	
Physical distancing practice				
54.	Is physical distancing being follower strictly in your workplace?	Nominal	1. Always<br>2. Most of the times<br>3. Sometimes<br>4. Rarely<br>5. Never	

55.	Is physical distancing being implemented in the places you visit like market, malls, departmental store	Nominal	1. Always<br>2. Most of the times<br>3. Sometimes<br>4. Rarely<br>5. Never	
56.	Do you think maintaining physical distancing is difficult in our setting?	Nominal	1. Strongly agree<br>2. Somewhat agree<br>3. Neither agree nor disagree<br>4. Somewhat disagree<br>5. Strongly disagree<br>6. Don't know/refused	
Practices in workplace (Skip if the answer to Q.no.10 is "7-Home maker")				
57.	How often you go to your workplace?	Nominal	1. Daily<br>2. Once in two-three days<br>3. Once in a week<br>4. More than a week<br>5. Never go to workplace (working from home)	End the interview if the answer is "Never"
58.	Do you wear face mask in your workplace?	Nominal	1. Always<br>2. Most of the times<br>3. Sometimes<br>4. Rarely<br>5. Never	
59.	Do you share your food while eating at workplace?	Nominal	1. Always<br>2. Most of the times<br>3. Sometimes<br>4. Rarely<br>5. Never	
60.	Is your temperature checked daily at your workplace?	Nominal	1. Always<br>2. Most of the times<br>3. Sometimes<br>4. Rarely<br>5. Never	

61.	Is hand sanitizer available at your workplace?	Nominal	1. Always<br>2. Most of the times<br>3. Sometimes<br>4. Rarely<br>5. Never	
62.	Does your workplace encourage self-reporting of symptoms?	Nominal	1. Yes<br>2. No<br>3. Not sure	
63.	Does your workplace offer paid leave if anyone is affected with COVID-19 or if any of the household members are affected with COVID-19?	Nominal	1. Yes<br>2. No<br>3. Not sure	End the interview if the answer is "No/Not sure"
64.	If yes, how many days of paid leave are given?	Numeric		
